# Curvature of the Spine

**Published:** 1867-09

**Authors:** 


### CURVATURE OF THE SPINE.

Dr. Earle reported three cases of curvature of the spine in children, which had been successfully treated by means of a modification of Dr. Taylor's apparatus, which the reporter exhibited and explained to the Society.

Dr. Danforth exhibited a singular, hard, chalky concretion, elipsoidal in shape, one and a half inches by an inch in diameter, which he had removed from under the chin of a female patient, 28 years of age. The tumor had existed twelve years.

